# Urinary bladder metastasis from breast cancer: a rare cause of hematuria

**DOI:** 10.1259/bjrcr.20190048

**Published:** 2020-02-12

**Authors:** Samuel Nguku Gitau, Allan Njau, Sitna Mwanzi

**Affiliations:** 1 Department of Radiology, Aga Khan University Hospital, Nairobi, Pakistan; 2 Department of Pathology, Aga Khan University Hospital, Nairobi, Pakistan; 3 Department of Oncology, Aga Khan University Hospital, Nairobi, Pakistan

## Abstract

Breast cancer is the most common cancer in women globally as well as in Kenya. The most common sites of metastases reported include the bones, liver and lung. Metastasis to the urinary bladder is relatively uncommon with only a few case reports in literature. It can therefore be easily overlooked as a cause of hematuria in these patients. We describe a rare case of a patient with breast cancer who presented with urinary bladder metastasis as a late complication of her illness.

## Case review

A 54-year-old female presented with 1 week history of self-discovered left breast lump in August 2010. The lump was painless with no skin or nipple changes and no axillary swelling. She was post-menopausal with three grown children. She was a lifelong non-smoker and did not take alcohol. There was no family history of cancer. Initial mammography and left breast ultrasound revealed a 1.4 × 0.9 cm irregular mass in the left upper outer quadrant. Biopsy of the lesion confirmed invasive lobular carcinoma that was estrogen receptor (ER) and progesterone receptor (PR) positive and human epidermal growth factor-2 (HER2) equivocal (2+), for which, HER2 Fluorescence *in situ* hybridization was negative. Bone scan showed two areas of increased uptake in the proximal and distal portion of the sternum suggestive of metastases. She had no other sites of metastases. In view of the oligometastatic disease, the patient was treated aggressively with modified radical mastectomy followed by adjuvant chemotherapy and radiotherapy. She was then started on endocrine therapy.

4 years later, she presented with increased bone pain which on CT scan and bone scan confirmed extensive bone metastases but no other sites of disease. She received palliative radiotherapy to the spine and treatment changed to everolimus and fulvestrant with denosumab. In 2016, she had skin nodules which were confirmed on biopsy to be invasive lobular carcinoma (ER/PR positive and HER2 negative). Therapy changed to single agent capecitabine.

**Figure 1.**
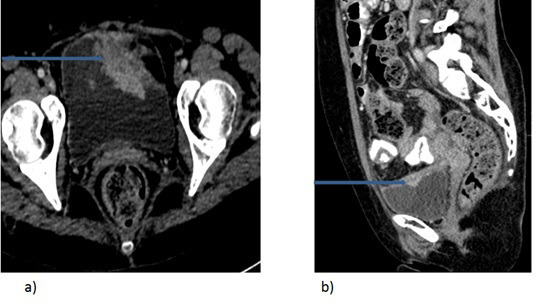
A 54-year-old female with breast cancer presenting with hematuria. Axial (a) and sagittal (b) CT images of the of the pelvis show enhancing irregular bladder wall thickening (arrows).

7 years after her initial diagnosis, the patient presented with abdominal fullness and hematuria. CT scan now showed extensive bladder wall thickening (Figure 1), new onset ascites and pleural effusion. Cystoscopy confirmed multiple tumor deposits in the bladder. Unfortunately, no sample was submitted for histology. Bilateral ureteric stents were inserted, ascitic and pleural drains inserted and patient regimen was changed. Her ECOG performance status remained one and received further lines of chemotherapy including gemcitabine and carboplatin, eribulin and nab-paclitaxel. 9 months after diagnosis of bladder metastases, her condition deteriorated and she was transitioned to best supportive care and she eventually succumbed to her illness.

## Discussion

While breast cancer is the most common malignancy in females, metastases to the urinary bladder are extremely rare. Approximately, 54 cases have been reported in literature to date.^1^ In several autopsy studies, the rate of bladder metastases from breast cancer range from 1.2 to 7%.^1^ Bladder metastases more commonly arise from tumors in the pelvic region such as the cervix, rectum and prostate.^2^ Breast cancer more commonly spreads to the liver, lung and bone.^2,3^

As in our patient, the most common histological subtype associated with bladder metastases is invasive lobular carcinoma even though invasive ductal carcinoma is the most common histological subtype of primary breast cancer.^1,2,4^ The postulated mechanisms of spread from the breast primary to the bladder include hematogenous spread of viable tumor emboli through pulmonary circulation without establishing lung metastases with subsequent bladder involvement. It is postulated that because invasive lobular carcinoma loses e-cadherin, this leads to more metastases as cell–cell adhesion is lost. In addition, lobular carcinoma being a serosal type cancer is likely to spread into the gastrointestinal system and genitourinary system.^2^

The most common presentation of patients with bladder metastases as with our case is gross hematuria. Other signs and symptoms include urinary frequency, incontinence, dysuria, lower abdominal pain and back pain.^5–7^ There is a recent report of an asymptomatic patient found to have bladder metastases on routine follow up CT scan 2 years after initial therapy for breast cancer.^8^

Imaging for patients suspected to have bladder metastases include bladder ultrasound, CT scan or MRI of the pelvis. Typically, bladder wall thickening is seen with or without hydronephrosis and hydroureter. Cystoscopy is mandatory for confirming the diagnosis. In majority of the case reports, cystoscopy revealed large tumor deposits as was found in our patient described above.^9^ Histology and immunohistochemistry including staining for ER and PR as well as HER2 is crucial in confirming the primary tumor from breast and to exclude a primary bladder cancer. Discordance in hormonal status in primary and metastatic sites has however been reported to be around 24 and 39%.^1^ More recent publications have not found additional information to that reported in previously cited literature.^3,4^

Therapies include local resection of the bladder tumor during cystoscopy, palliative radiotherapy and systemic therapy. Ureteric stenting may also be indicated for those with hydronephrosis and renal dysfunction. It has been reported that survival is short after diagnosis of bladder metastases.^10^

## Conclusion

Metastases to urinary bladder from breast cancer is a rare occurrence but should be considered for breast cancer patients presenting with urinary symptoms, in particular hematuria.

## Leaning points

1. Although rare, urinary bladder metastasis from breast cancer should be considered for patients presenting with urinary symptoms, in particular hematuria.
2. The most common histological subtype associated with bladder metastases is invasive lobular carcinoma.
3. Cystoscopy is recommended for patients with invasive lobular carcinoma presenting with hematuria to exclude bladder metastasis.
